# Adverse pregnancy outcomes on the risk of overweight offspring: a population-based retrospective study in Xiamen, China

**DOI:** 10.1038/s41598-020-58423-7

**Published:** 2020-01-31

**Authors:** Yin-ling Chen, Li-li Han, Xiu-lin Shi, Wei-juan Su, Wei Liu, Li-ying Wang, Pei-ying Huang, Ming-zhu Lin, Hai-qu Song, Xue-jun Li

**Affiliations:** 1 Department of Endocrinology and Diabetes, the First Affiliated Hospital of Xiamen University, Xiamen, China; 2 Fujian Medical University, Fuzhou, China

**Keywords:** Diabetes, Obesity

## Abstract

The growth trajectory of Chinese preschoolers still remains unclear. Our objective was to determine whether there was an association between adverse pregnancy outcomes and overweight offspring. We analyzed population-based retrospective cohort data from the Medical Birth Registry of Xiamen, which comprised 33,157 children examined from 1 to 6 years of age. Longitudinal analyses were used to evaluate the growth trajectories of offspring body mass index (BMI). Multivariate logistic regression was used to assess the effects of two adverse pregnancy outcomes, gestational diabetes mellitus (GDM) and being large-for-gestational age (LGA), on childhood overweight. Offspring of mothers with GDM and LGA has a higher annual BMI z-score from 1 to 6 years of age (all *P* < 0.05). But, a higher annual BMI z-score was only observed in children aged 1–5 years in models 1–3. Overall BMI z-score of offspring aged 1–6 who were born to mothers with GDM and LGA were also higher in models 1–3 (all *P* < 0.05). Additionally, offspring of mothers with GDM and LGA had a higher risk for overweight in model 1, from 1 to 6 years of age (odds ratio (OR), 1.814; 95% confidence interval (CI), 1.657–1.985; *P* < 0.0001). However, this association was attenuated after adjusting for maternal pre-pregnancy BMI (OR, 1.270; 95% CI, 0.961–1.679; *P* = 0.0930). Offspring of mothers with GDM and LGA had a higher BMI z-score and increased risk for overweight. Indeed, intrauterine exposure to maternal GDM and LGA could bias offspring to overweight, whereas maternal pre-pregnancy BMI may play a key role in offspring overweight for children born to mothers with GDM and LGA.

## Introduction

Worldwide, there are 155 million children aged 5–17 years who are overweight or obese^1^. However, the prevalence of overweight among children in China aged 0–6 years increased from 6.5% in 2002 to 8.4% in 2013^2^. Consequently, identifying risk factors is necessary for early intervention and prevention of childhood overweight or obesity. It has been reported that exposure to hyperglycaemia in utero may increase the risk for lifelong obesity due to gestational diabetes mellitus (GDM)^3^. GDM is characterised by impaired glucose intolerance with first recognition during pregnancy and is linked to substantial rates of perinatal or maternal complications. Indeed, GDM can affect many common pregnancy outcomes, and influencing 1–28 percent of pregnancies in 173 countries^4,5^. The incidence of GDM is increasing worldwide, particularly in China^6^, and this is related to adverse pregnancy outcome such as large-for-gestational age (LGA) birth complications^7^. Compared with non-diabetic women, LGA infants born to women with GDM have distinctly elevated fat masses^8^. But, disproportionate intrauterine growth can lead to an increased abdominal perimeter^9^.

How GDM and LGA during pregnancy affects childhood growth is not well understand. Only three studies have examined the effects of both GDM and LGA on childhood overweight or obesity^10–12^. In addition, to our knowledge, there are no data available on childhood body mass index (BMI) z-scores in the Chinese population. These data are necessary to understand the effect of GDM and LGA pregnancies on adverse childhood health outcomes including overweight or obesity, in this population.

Therefore, we conducted this study to track BMI z-score from infancy to early childhood (1–6 years) in a large population and to thereby determine the association between BMI z-score and offspring overweight with mother with GDM and LGA.

## Results

### Study participants characteristics

**Figure 1:**
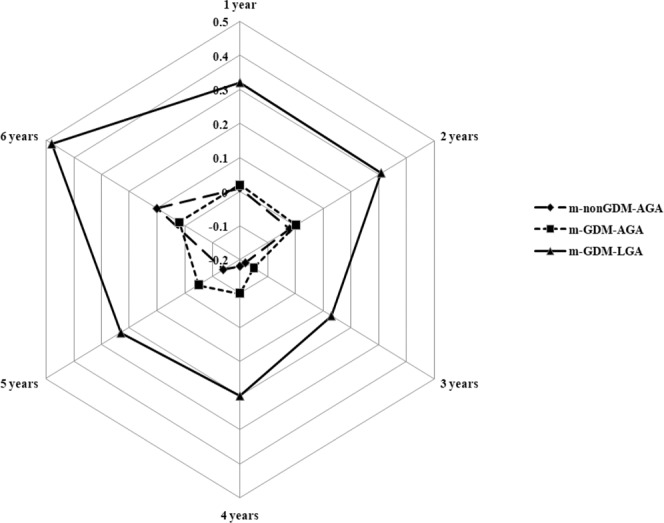
The annual BMI z-score for offspring who were born to m-nonGDM-AGA, m-GDM-AGA, and m-GDM-LGA pregnancy aged 1 to 6 years without adjustment for factors.

Our study population comprised 33,157 mother-child pairs, of which 26,379 mothers qualified as non-GDM and appropriate-for-gestational age (AGA) (m-nonGDM-AGA), 5,179 mothers qualified as GDM and AGA (m-GDM-AGA), and 1,599 mothers qualified as GDM and LGA (m-GDM-LGA) (Table 1). Offspring who were born from m-GDM-LGA pregnancies were more likely to have higher BMI z-score from 1–6 years of age (all *P* < 0.05) (Fig. 1). In addition, compared with m-nonGDM-AGA and m-GDM-AGA mothers, m-GDM-LGA mothers were more likely to have a higher pre-pregnancy BMI (*P* < 0.001). Although maternal age, blood pressure, and oral glucose tolerance test (OGTT) value among the three groups were all significantly different, the m-GDM-LGA group was slightly high compared to the other two groups. More than half of the babies were male in three groups (50.77%, 50.88%, and 63.79%, respectively). Most offspring were fed with a mixture of breast milk and formula within the first six months. The proportion of offspring that were exclusively breastfed was the least frequently used feeding method (*P* = 0.0319).

**Table 1:** Characteristics of mother exposed to LGA or GDM on child obesity.

Variable	Available n	Exposed to m-nonGDM-AGA (n = 26379)	Exposed to m-GDM-AGA (n = 5179)	Exposed to m-GDM-LGA (n = 1599)	*P* value
**Maternal characteristics**					
Maternal age before pregnancy, mean(SD), y	32946	27.9 (3.8)	29.9 (4.3)	30.5 (4.4)	<0.0001
Gestational age at delivery, mean(SD), week	33105	39.0 (3.4)	38.5 (5.3)	38.6 (4.7)	0.5340
Pre-pregnancy BMI, mean(SD), kg/m^2^	33082	20.6 (2.6)	21.8 (3.1)	23.0 (3.2)	<0.0001
Pre-pregnancy BMI category, n (%)	33082				<0.0001
<18.5		5649 (21.5)	663 (12.8)	75 (4.7)	
18.5–23.9		17922 (68.1)	3361 (65.1)	961 (60.1)	
24.0–27.9		2392 (9.1)	945 (18.3)	446 (27.9)	
≥28.0		351 (1.3)	201 (3.9)	116 (7.3)	
Gestational weight gain, mean(SD), kg	2378	1.8 (2.0)	2.7 (2.9)	2.5 (2.8)	<0.0001
Education, year (%)	30162				<0.0001
≤ 9		5237 (21.8)	1135 (24.1)	368 (24.9)	
> 9		18742 (78.2)	3572 (75.9)	1108 (75.1)	
Mean systolic pressure, mean(SD), mmHg	18907	107.1 (10.5)	109.6 (10.9)	109.9 (11.6)	<0.0001
Mean diastolic pressure, mean(SD), mmHg	18903	65.5 (7.8)	67.1 (8.2)	67.2 (8.3)	<0.0001
Fasting plasma glucose level, mean(SD), mmol/L	33157	4.4 (0.3)	4.8 (0.5)	5.0 (0.5)	<0.0001
1-h plasma glucose level, mean(SD), mmol/L	33157	7.4 (1.3)	9.9 (1.5)	10.0 (1.5)	<0.0001
2-h plasma glucose level, mean(SD), mmol/L	33157	6.3 (1.0)	8.4 (1.5)	8.5 (1.5)	<0.0001
**Child characteristics**					
Boy, %	17048	13393 (50.77)	2635 (50.88)	1020 (63.79)	<0.001
Birth weight, kg	33157	3.2 (0.3)	3.1 (0.4)	3.7 (0.3)	<0.001
Mode of infant feeding within the first 6 months, %	31409				0.0319
Exclusive formula feeding		3864 (15.48)	838 (17.02)	223 (14.60)	
Exclusive breastfeeding		2513 (10.07)	467 (9.48)	142 (9.30)	
Mixed breast and formula		18580 (74.45)	3620 (73.50)	1162 (76.10)	
BMI z-scores for age, mean (SD),					
1 year	32371	0.01 (0.91)	0.02 (0.92)	0.32 (0.93)	<0.0001
2 years	22743	−0.02 (0.87)	0.003 (0.89)	0.31 (0.87)	<0.0001
3 years	16317	−0.18 (0.92)	−0.15 (0.94)	0.13 (0.95)	<0.0001
4 years	7250	−0.18 (0.92)	−0.10 (0.95)	0.20 (0.93)	<0.0001
5 years	3271	−0.14 (0.93)	−0.05 (1.00)	0.23 (0.99)	<0.0001
6 years	345	−0.10 (1.03)	0.02 (1.04)	0.48 (0.98)	0.038

GDM, gestational diabetes mellitus; LGA, large for gestational age; AGA, appropriate for gestational age; m, mother; BMI, body mass index.

### Effects of m-GDM-LGA on offspring BMI z-score

All pregnant women were divided into three groups according to maternal GDM and gestational age status, as shown in Table 2: m-nonGDM-AGA, m-GDM-AGA, and m-GDM-LGA. In models 1, 2, and 3, offspring who were born to m-GDM-LGA pregnancy were more likely to have higher BMI z-score from 1 to 5 years of age, compared with offspring who were born to m-nonGDM-AGA pregnancy or m-GDM-AGA pregnancy (all *P* < 0.05). However, no significant differences were observed for offspring BMI z-score at 6 years of age, even after adjusting for pre-pregnancy BMI and other covariates (all *P* > 0.05).

**Table 2:** Comparison of z-scores for BMI according to maternal GDM and LGA in different age.

BMI z-score for age	Exposed to m-nonGDM-AGA	Exposed to m-GDM-AGA	Exposed to m-GDM-LGA	*P* value
Age 1 year				
Model 1	0.01 (0.01)	0.01 (0.02)	0.31 (0.03)	<0.0001
Model 2	0.15 (0.02)	0.16 (0.02)	0.45 (0.03)	<0.0001
Model 3	0.16 (0.02)	0.13 (0.02)	0.39 (0.03)	<0.0001
Age 2 year				
Model 1	−0.01 (0.01)	0.01 (0.02)	0.33 (0.03)	<0.0001
Model 2	0.06 (0.02)	0.08 (0.02)	0.40 (0.03)	<0.0001
Model 3	0.06 (0.02)	0.05 (0.02)	0.32 (0.03)	<0.0001
Age 3 year				
Model 1	−0.15 (0.01)	−0.12 (0.02)	0.16 (0.04)	<0.0001
Model 2	−0.11 (0.02)	−0.09 (0.03)	0.19 (0.04)	<0.0001
Model 3	−0.10 (0.02)	−0.12 (0.03)	0.09 (0.04)	<0.0001
Age 4 year				
Model 1	−0.15 (0.02)	−0.04 (0.04)	0.21 (0.06)	<0.0001
Model 2	−0.12 (0.03)	−0.02 (0.04)	0.23 (0.06)	<0.0001
Model 3	−0.12 (0.03)	−0.07 (0.04)	0.11 (0.06)	0.0002
Age 5 year				
Model 1	−0.11 (0.03)	0.03 (0.06)	0.18 (0.09)	0.0003
Model 2	−0.10 (0.03)	0.04 (0.06)	0.19 (0.09)	0.0003
Model 3	−0.10 (0.03)	−0.01 (0.06)	0.07 (0.09)	0.0447
Age 6 year				
Model 1	−0.07 (0.12)	0.02 (0.19)	0.24 (0.25)	0.4094
Model 2	−0.08 (0.13)	0.03 (0.20)	0.28 (0.26)	0.3165
Model 3	−0.11 (0.14)	0.02 (0.20)	0.21 (0.26)	0.3645

Data are showed as mean (SE).

Model 1: adjusted for sex, maternal age, education, and infant feeding;

Model 2: adjusted for covariates in Model 1 + maternal gestational weight gain;

Model 3: adjusted for covariates in Model 2 + maternal pre-pregnancy body mass index.

GDM, gestational diabetes mellitus; LGA, large for gestational age; AGA, appropriate for gestational age; m, mother; BMI, body mass index.

**Figure 2:**
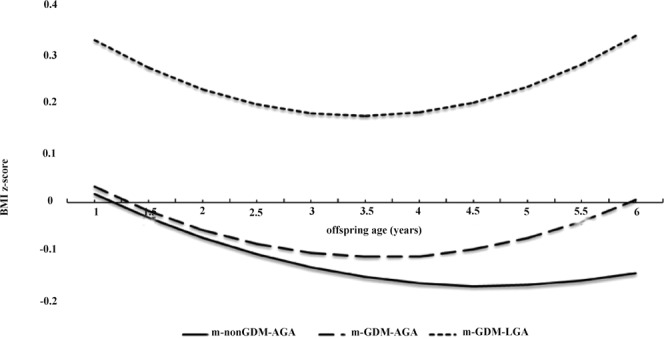
Growth trajectory of BMI z-score for offspring who were born to m-nonGDM-AGA, m-GDM-AGA, and m-GDM-LGA pregnancy aged 1 to 6 years with mixed model. LGA, large for gestational age; AGA, appropriate for gestational age; GDM, gestational diabetes mellitus; m, mother.

Our mixed model showed that offspring exposed to m-GDM-LGA pregnancy had the highest BMI Z-score trajectory compared with those exposed to m-GDM-AGA and m-nonGDM-AGA pregnancy in an unadjusted model (Fig. 2).

### Effects of m-GDM-AGA on offspring BMI z-score and overweight

In this longitudinal analysis, m-GDM-AGA was significantly associated with a higher BMI z-score (models 1, 2, and 3) and overweight (models 2 and 3) across the time span of 1–6 years of age, even after adjusting for maternal pre-pregnancy BMI (Table 3). The offspring who were born to m-GDM-AGA pregnancy had 1.052-fold higher odds of becoming overweight from 1 to 6 years (OR, 1.052; 0.985–1.122; *P* = 0.1292) after adjusting for model 1 covariates, 1.528-fold higher odds after adjusting for model 2 (model 1 plus maternal gestational weight gain, *P* < 0.0001), and 1.480-fold higher odds after adjusting for model 3 covariates (model 2 plus pre-pregnancy BMI, *P* < 0.0001).

**Table 3:** The joint effects of GDM and LGA on BMI z-score and risk of overweight.

Outcome	m-GDM-AGA				m-GDM-LGA			
	BMI z-score		Overweight		BMI z-score		Overweight	
	Absolute changes in z-score estimate (95% CI)	*P* value	OR (95% CI)	*P* value	Absolute change in z-score estimate (95% CI)	*P* value	OR (95% CI)	*P* value
Model 1	0.023 (0.003–0.042)	0.0212	1.052 (0.985–1.122)	0.1292	0.315 (0.283–0.347)	<0.0001	1.814 (1.657–1.985)	<0.0001
Model 2	0.107 (0.049–0.164)	0.0003	1.528 (1.283–1.818)	<0.0001	0.253 (0.162–0.344)	<0.0001	1.366 (1.035–1.802)	0.0273
Model 3	0.087 (0.030–0.145)	0.0028	1.480 (1.243–1.764)	<0.0001	0.218 (0.128–0.309)	<0.0001	1.270 (0.961–1.679)	0.0930

Model 1: adjusted for maternal age, education, and infant feeding, sex; Model 2: adjusted for covariates in Model 1 + maternal gestational weight gain; Model 3: adjusted for covariates in Model 2 + maternal pre-pregnancy body mass index. GDM, gestational diabetes mellitus; LGA, large for gestational gae; AGA, appropriate for gestational age; m, mother; BMI, body mass index; OR, odds ratio; CI, confidence intervals.

### Effects of m-GDM-LGA on offspring BMI z-score and overweight

Offspring who were born to m-GDM-LGA pregnancy were more likely to have higher BMI z-score (models 1, 2, and 3) and become overweight (models 1 and 2) in early childhood (Table 3). After adjusting for model covariates, offspring who were born to m-GDM-LGA pregnancy had 1.814-fold higher odds of becoming overweight from 1 to 6 years of age (OR, 1.814; 1.657–1.985; *P* < 0.0001). 1.366-fold higher odds after adjusting for model 2 (model 1 plus maternal gestational weight gain) covariates (*P* = 0.0273), and 1.270-fold higher odds after adjusting for model 3 (model 2 plus pre-pregnancy BMI) covariates (*P* = 0.0930).

## Discussion

To our knowledge, this is a large study to research association between GDM and LGA and preschool-aged children overweight after adjusting for covariates, and the first to associate BMI z-score with children overweight in preschool-aged children in China. We observed that offspring born to m-GDM-LGA pregnancy showed BMI z-score acceleration from 5 to 6 years of age compared with offspring born to m-GDM-AGA pregnancy. Meanwhile, annual BMI z-score had accelerated from 3 to 6 years of age. These results are consistent with a previous study that found BMI standard-deviation score increased with age, with particularly high acceleration during preschool years^13^. Several studies have also reported that a relationship exists between adolescent and adult overweight or obesity and high BMI during childhood^14–16^. In addition, only 20 percent of young children who are overweight or obese will become a normal weight during adolescence. Previous studies have tracked BMI trajectories, found that high-growth BMI trajectory groups diverge from stable BMI trajectory groups starting at around 3 years of age^17,18^. These results are consistent with our analysis.

Offspring born to m-GDM-LGA pregnancy retained higher BMI z-score during preschool years and had a higher risk of overweight compared with offspring born to m-GDM-AGA pregnancy after adjusting for covariates. However, the association between m-GDM-LGA pregnancy and offspring overweight was attenuated after adjusting for pre-pregnancy BMI. Several studies on offspring born to a GDM pregnancy have also observed similar results on overweight^19,20^. In contrast, considering that pre-pregnancy BMI may significantly affect GDM^21^, offspring who were born to a GDM pregnancy cannot be directly compared to mother who have a higher pre-pregnancy BMI, on account of different methodology. Unexpectedly, there was no difference in BMI between LGA and non-LGA offspring born to GDM pregnancy in a study of 6 to 7-year-old infants^11^.

With regard to childhood overweight or obesity, it is becoming increasingly clear that GDM can affect foetal metabolism and growth, which results in higher adiposity in the offspring^1^. The ‘fetal programming theory’ emphasizes the significance of long term effect of suboptimal intrauterine exposure on offspring^22^. Hyperglycaemia in uterus can result in fetal overnutrition and elevated oxidative stress that induces pro-inflammatory response^23^, methylation modifications^24^, insulin secretion increasing^25^, which can lead to hypothalamus epigenetic and neurohormonal changes^26^, and high adiposity at birth^3^. Ultimately, these may lead to offspring overweight or obesity later in life^1^. A number of studies have investigated the association between GDM and offspring overweight or obesity^27–29^, but have inconsistent results. A Swedish study suggested birth weight only played a minimal role in the relationship between GDM and offspring overweight or obesity. Overall, maternal GDM may predispose offspring to overweight or obesity later in life via mechanisms beyond excessive fetal growth, as illustrated by birth weight.

The major strength of this population-based study is a large sample size. Moreover, detailed information was obtained on pre-pregnancy BMI, which was no adequately adjusted for in previous studies. However, this study also has several limitations. Firstly, we did not have BMI z-score data for every offspring from 1 to 6 years of age. Secondly, study participants were all from the Chinese population, and therefore future studies among other ethnicities are needed. Thirdly, data on risk factors for offspring overweight or obesity such as maternal smoking or alcohol consumption would have improved the study.

In conclusion, this is a large population-based retrospective study on Chinese mother-child pairs. Offspring of mothers with GDM and LGA had a higher BMI z-score and increased risk of overweight from 1 to 6 years of age. However, the later association was attenuated after adjusting for maternal pre-pregnancy BMI, suggesting maternal pre-pregnancy BMI may play a key role in offspring overweight for children born to mothers with GDM and LGA. Therefore, we should focus on maternal pre-pregnancy weight to reduce the prevalence of childhood overweight or obesity. Further researches needed to expand these results to other populations, and to identify the underlying biological mechanisms.

## Methods

### Study design

We conducted a population-based retrospective study using the healthcare records data from the Medical Birth Registry in Xiamen (MBRX), China, between January 2011 and March 2018. This was a registration system established in 2007 in Xiamen based on a compulsory notification of all live and stillbirths from 12 weeks’ gestation onward. This study was approved by the ethics committee of the First Affiliated Hospital of Xiamen University (KYH2018–007) and conducted in accordance with the rules of the Declaration of Helsinki of 1975, revised in 2013. Informed consent was not required because this was a retrospective study.

### Data sources of MBRX

All women in Xiamen are registered at their community health centres when they get pregnant, and were then referred to a secondary hospital or a tertiary hospital for healthcare from the 32nd gestational week till delivery. All children were given health examinations every year from birth (<3 days after birth) until the age of 6. Women and children were linked by individual record linkages to the Xiamen citizen health information system using the person-unique identification number assigned to each Xiamen citizen. Every child was also linked to his/her biological mother’s maternal identification number.

### Study population

A total of 33,157 mother-child pair healthcare records were available. All women over the age of 18 performed a 75-g OGTT between 24 and 28 weeks of gestational in this study. Eligibility criteria were as follows: (1) an OGTT was conducted between 24 and 28 weeks of gestation; (2) gestational age at delivery ≥37 weeks, with no major neonatal malformations or fetal/neonatal death; and (3) the offspring was followed-up through 6 years of age. Exclusion criteria included: (1) missing a mother’s weight or height information at pre-pregnancy; (2) medical history of diabetes (diagnosed before the index pregnancy); and (3) fasting glucose level ≥7.0 mmol/L before 12 gestational weeks, as this could indicate an under-diagnosed diabetes cases prior to pregnancy.

### Maternal and offspring characteristics

Information from MBRX on maternal factors included age, education, weight in 12 weeks before of pregnancy, occupation, first visit date, numbers of pregnancy/infants, last menstrual period, expected delivery date, smoking habits, drunk status, medical history, family history of disease, hypertension history, pregnancy reactions, as well as labour status. Furthermore, GDM, gestational weight gain, gestational age at delivery, height, weight, blood pressure, fasting glucose, gynaecological examinations, ultrasonography, gestational diabetes screening results, other lab tests results, complications during pregnancy, and pregnancy outcomes were also included in the MBRX system.

MBRX also included information from newborns to preschool-aged children on date of birth, sex, gestational week of birth, weight, Apgar score, names of the child and his/her parents, family history of diseases, feeding modalities (exclusive breast feeding, mixed breast and formula feeding, and exclusive formula feeding) during the first 6 months, date of examination, weight, height, number of teeth, and blood pressure.

### Offspring measurements and data transformations

During each health examination, each child’s height and weight was measured by a trained clinician. Body weight was measured in kilograms using regularly calibrated electronic scales.

Children were classified into three groups according to GDM and gestational age status during pregnancy: 1) children born to m-nonGDM-AGA; 2) children born to m-GDM-AGA; and 3) children born to m-GDM-LGA. Body mass index (BMI) was calculated as weight (kg) / height^2^ (m^2^). BMI z-score for age was used to present the trajectory tracking of offspring BMI. We calculated sex-adjusted and age-adjusted z-score of childhood BMI referred to Chinese reference growth charts^30^. Childhood overweight or obesity were also defined by age-specific and sex-specific Chinese criteria^30^.

### Variables definition

#### Gestational diabetes mellitus

GDM cases were identified between 24 and 28 weeks’ gestation by conducting OGTT, which period is considered as the optimal period to make GDM diagnosis. According to the 2014 National Health and Family Planning Commission of the People’s Republic of China criteria, after a 75 g glucose load, pregnant women would be considered to have GDM if one of the following plasma glucose values was met or exceeded: 0 hour, 5.1 mmol/L; 1 hour, 10.0 mmol/L; or 2 hours, 8.5 mmol/L^6^. Even if the test was performed after 28 weeks, it was still considered valid.

### Large for gestational age

LGA was defined as birth weight was above 90 percentile for gestational age, according to gestational age and gender-specific intergrowth-21^st^ curves^31^.

### Statistical analysis

Mean±SD was showed for continuous variables, and discontinuous variables were presented as n (%). To evaluate our hypothesis that GDM and LGA were associated with offspring BMI growth trajectories, we performed several analysis. Firstly, mean BMI z-score were visually compared in yearly time intervals between m-nonGDM-AGA, m-GDM-AGA, and m-GDM-LGA. Secondly, the growth trajectories of offspring BMI were established by the longitudinal analyses that fitted flexible and smooth curves with a random-effects model^32^. The square model was used in BMI z-score points. The values of children from the GDM and LGA group and from children in the non-GDM and LGA group were modelled as shown below: Y = Intercept + β_0ij_ + β_1ij_ (age) + β_2ij_ (age)^2^. In addition, considering the previous studies and clinical relevance^33^, we assessed the joint effect of GDM and pre-pregnancy BMI (a major risk factor for offspring overweight or obesity), using a model with a combination of GDM (yes, no) and BMI categories^34^ (BMI < 23.9 kg/m^2^, 24–27.9 kg/m^2^, or ≥28 kg/m^2^) at 6 years of age. Thirdly, a logistic regression model was used to access the significance of offspring overweight between m-GDM-AGA and m-GDM-LGA groups. Three multivariable-adjusted models were included in this study. Model 1 adjusted for offspring sex, maternal age, education, and infant feeding; model 2 adjusted for the variables in model 1 plus maternal gestational weight gain; model 3 adjusted for the variables in model 2 plus maternal pre-pregnancy BMI. Statistical significance was two-tailed with a P-value < 0.05. All calculations were carried out using SAS 9.4 (SAS Institute Inc, Cary, North Carolina, USA).

## Informed consent from participants

Informed consent of this retrospective study was waived by the ethics committee of the First Affiliated Hospital of Xiamen University (KYH2018–007).

## Data Availability

The datasets generated during and/or analysed during the current study are available from the corresponding author on reasonable request.

## References

[1] Gillman MW, Ludwig DS (2013). How early should obesity prevention start?. N. Engl. J. Med..

[2] Huang Y (2017). Effect of maternal glycemia and weight status on offspring birth measures and BMI-z among Chinese population in the first year. Sci. Rep..

[3] Lawlor DA (2013). The Society for Social Medicine John Pemberton Lecture 2011. Developmental overnutrition–an old hypothesis with new importance?. Int. J. Epidemiol..

[4] Zhou J (2016). Potential Role of Hyperglycemia in Fetoplacental Endothelial Dysfunction in Gestational Diabetes Mellitus. Cell Physiol. Biochem..

[5] Jiwani A (2012). Gestational diabetes mellitus: results from a survey of country prevalence and practices. J. Matern. Fetal Neonatal Med..

[6] Yan B (2019). High, but stable, trend in the prevalence of gestational diabetes mellitus: A population-based study in Xiamen, China. J. Diabetes Investig..

[7] Group HSCR (2008). Hyperglycemia and adverse pregnancy outcomes. N. Engl. J. Med..

[8] Durnwald C, Huston-Presley L, Amini S, Catalano P (2004). Evaluation of body composition of large-for-gestational-age infants of women with gestational diabetes mellitus compared with women with normal glucose tolerance levels. Am. J. Obstet. Gynecol..

[9] Hammoud NM (2013). Fetal growth profiles of macrosomic and non-macrosomic infants of women with pregestational or gestational diabetes. Ultrasound Obstet. Gynecol..

[10] Kaul P (2019). Association between maternal diabetes, being large for gestational age and breast-feeding on being overweight or obese in childhood. Diabetologia.

[11] Boney CM, Verma A, Tucker R, Vohr BR (2005). Metabolic syndrome in childhood: association with birth weight, maternal obesity, and gestational diabetes mellitus. Pediatrics.

[12] Hammoud NM (2018). Long-term BMI and growth profiles in offspring of women with gestational diabetes. Diabetologia.

[13] Geserick M (2018). Acceleration of BMI in Early Childhood and Risk of Sustained Obesity. N. Engl. J. Med..

[14] Ward ZJ (2017). Simulation of Growth Trajectories of Childhood Obesity into Adulthood. N. Engl. J. Med..

[15] Mead E, Batterham AM, Atkinson G, Ells LJ (2016). Predicting future weight status from measurements made in early childhood: a novel longitudinal approach applied to Millennium Cohort Study data. Nutr. Diabetes.

[16] Cunningham SA, Kramer MR, Narayan KM (2014). Incidence of childhood obesity in the United States. N. Engl. J. Med..

[17] Pryor LE (2011). Developmental trajectories of body mass index in early childhood and their risk factors: an 8-year longitudinal study. Arch. Pediatr. Adolesc. Med..

[18] Stuart B, Panico L (2016). Early-childhood BMI trajectories: evidence from a prospective, nationally representative British cohort study. Nutr. Diabetes.

[19] Catalano PM (2009). Perinatal risk factors for childhood obesity and metabolic dysregulation. Am. J. Clin. Nutr..

[20] Gillman MW, Rifas-Shiman S, Berkey CS, Field AE, Colditz GA (2003). Maternal gestational diabetes, birth weight, and adolescent obesity. Pediatrics.

[21] Nilsson C, Carlsson A, Landin-Olsson M (2014). Increased risk for overweight among Swedish children born to mothers with gestational diabetes mellitus. Pediatr. Diabetes.

[22] Gillman MW (2004). A Life Course Approach to Obestidy..

[23] Westermeier F, Saez PJ, Villalobos-Labra R, Sobrevia L, Farias-Jofre M (2014). Programming of fetal insulin resistance in pregnancies with maternal obesity by ER stress and inflammation. Biomed. Res. Int..

[24] Ruchat SM (2013). Gestational diabetes mellitus epigenetically affects genes predominantly involved in metabolic diseases. Epigenetics.

[25] Wattez JS (2013). Perinatal nutrition programs the hypothalamic melanocortin system in offspring. Horm. Metab. Res..

[26] Remmers F, Delemarre-van de Waal HA (2011). Developmental programming of energy balance and its hypothalamic regulation. Endocr. Rev..

[27] Kim SY, England JL, Sharma JA, Njoroge T (2011). Gestational diabetes mellitus and risk of childhood overweight and obesity in offspring: a systematic review. Exp. Diabetes Res..

[28] Kim SY, Sharma AJ, Callaghan WM (2012). Gestational diabetes and childhood obesity: what is the link?. Curr. Opin. Obstet. Gynecol..

[29] Philipps LH (2011). The diabetic pregnancy and offspring BMI in childhood: a systematic review and meta-analysis. Diabetologia.

[30] Li H, Ji CY, Zong XN, Zhang YQ (2009). [Body mass index growth curves for Chinese children and adolescents aged 0 to 18 years]. Zhonghua Er Ke Za Zhi.

[31] Villar J (2014). International standards for newborn weight, length, and head circumference by gestational age and sex: the Newborn Cross-Sectional Study of the INTERGROWTH-21st Project. Lancet.

[32] Laird NM, Ware JH (1982). Random-effects models for longitudinal data. Biometrics.

[33] Catalano PM (2012). The hyperglycemia and adverse pregnancy outcome study: associations of GDM and obesity with pregnancy outcomes. Diabetes Care.

[34] Zhou BF (2002). Coorperative Meta-Analysis Group Of China Obesity Task, [Predictive values of body mass index and waist circumference to risk factors of related diseases in Chinese adult population]. Zhonghua Liu Xing Bing. Xue Za Zhi.

